# Spironolactone in Alcohol Use Disorder (SAUD): Introduction to an ongoing double-blind, placebo-controlled, ascending dose, Phase 1b study

**DOI:** 10.1192/j.eurpsy.2024.819

**Published:** 2024-08-27

**Authors:** A. Leko, M. Farokhnia, S. T. Weiss, C. A. Blake, L. A. Farinelli, L. Leggio

**Affiliations:** ^1^Translational Addiction Medicine Branch, NIDA and NIAAA, Baltimore and Bethesda, United States; ^2^Department of Psychiatry and Psychotherapy, Semmelweis University, Budapest, Hungary

## Abstract

**Introduction:**

Efforts are critically needed to increase the armamentarium of options that clinicians can use to treat patients with alcohol use disorder (AUD). Numerous preclinical studies support the hypothesis that mineralocorticoid receptor (MR) pharmacological antagonism may represent a novel and promising treatment for AUD. Namely, the non-selective MR antagonist spironolactone dose-dependently decreased 1) the intake of alcohol in mice in a model of alcohol binge drinking procedure and 2) alcohol self-administration in dependent and non-dependent rats (Farokhnia, Rentsch, Choung *et al.,* Mol Psychiatry 2022; 27(11):4642-4652). Furthermore, two U.S.-based independent human pharmacoepidemiologic studies utilizing electronic health records data showed that patients treated with spironolactone for any indication reduced their weekly alcohol use in a primary care-type medical setting (Palzes *et al.,* Neuropsychopharmacology 2021; 46(12):2140-2147) and Alcohol Use Disorders Identification Test-Consumption (AUDIT-C) score in a Veterans Affairs medical setting (Farokhnia, Rentsch, Choung *et al.,* 2022*;* 27(11):4642-4652). In both studies, spironolactone-treated patients were compared to matched ones without spironolactone prescription using propensity score matching.

**Objectives:**

We are conducting a Phase 1b human study to assess the pharmacokinetics and pharmacodynamics of spironolactone-alcohol co-administration and testing the safety and tolerability of spironolactone, alone and combined with alcohol in individuals with AUD.

**Methods:**

Spironolactone in Alcohol Use Disorder (SAUD) is a double-blind, placebo-controlled, randomized, within-subject, ascending dose study with spironolactone (0, 100, 200, 400 mg/day) PO for 5 days to reach steady-state, followed by oral fixed-dose alcohol administration aimed at reaching a blood alcohol level of approximately 0.08 %. Our sample consists of 12 adults diagnosed with AUD.

**Results:**

The primary endpoint is to measure spironolactone and alcohol PK during concomitant administration. Our secondary endpoints are 1) assessment of subjective and cognitive effects of acute alcohol administration during concomitant spironolactone treatment; 2) number and severity of adverse events (AEs) experienced, compared between placebo (0 mg/day) and all three spironolactone doses; 3) PK characteristic of spironolactone active metabolites, canrenone, 7-α-thiomethylspirolactone (TMS) and 6β-hydroxy-7α-thiomethylspirolactone (HTMS), before and after administration of alcohol. Recruitment is underway.

**Conclusions:**

The above-mentioned preclinical and clinical evidence suggest that spironolactone may be repurposed for the treatment of AUD. Our Phase 1b study is a key step before moving to larger efficacy trials.

**Disclosure of Interest:**

None Declared

